# Acute Compartment Syndrome of the Hand Following Multiple Metacarpal Fractures: Current Concepts

**DOI:** 10.7759/cureus.75714

**Published:** 2024-12-14

**Authors:** Grigorios E Kastanis, Mikela-Rafaella Siligardou, Constantinos Chaniotakis, Ioannis M Stavrakakis, Petros Kapsetakis

**Affiliations:** 1 Orthopedic Department, Venizeleio General Hospital, Heraklion, GRC

**Keywords:** compartment syndrome, fasciotomy, hand, metacarpal fractures, oedema

## Abstract

Metacarpal fractures are among the most common injuries seen in the emergency department, accounting for 17.2% of all adult fractures and more than 30% of all hand injuries. The majority of these cases are stable, and conservative treatment involving closed reduction and immobilization typically yields good clinical and functional outcomes. However, acute compartment syndrome (ACS) of the hand is a critical condition that can result in irreversible changes to the neurovascular supply, leading to ischemia, permanent loss of hand function, or even amputation. The purpose of this study is to present two patients with multiple metacarpal fractures following high-energy trauma who developed hand compartment syndrome (HCS), emphasizing the need for heightened suspicion of this injury among emergency department physicians to ensure prompt diagnosis and treatment. We strongly recommend hospitalization for patients with multiple metacarpal fractures in cases of high-energy trauma, particularly if they lack the ability to obtain prompt follow-up or return to the emergency department, to closely monitor the hand for potential development of HCS.

## Introduction

Compartment syndrome (CS) is characterized by increased interstitial tissue pressure within an enclosed fascial compartment, leading to decreased tissue oxygenation, and it may present as acute or intermittent/recurrent [1,2]. Acute compartment syndrome (ACS) of the hand, although it occurs less frequently, is not rare and can lead to adverse consequences [3,4]. In a recent systematic review by Alsaedi et al., the incidence of fracture-related hand compartment syndrome (HCS) was reported to be about 5.4% [5].

Metacarpal fractures are frequent injuries, representing around 30% to 40% of all hand fractures [3]. Isolated closed metacarpal fractures typically result from low-energy injuries, and conservative treatment provides good functional outcomes [4]. In contrast, multiple closed metacarpal fractures are often caused by high-energy injuries, such as traffic accidents and crush injuries to the hand (due to high compressive force), accompanied by significant soft tissue swelling from large hematomas in the interosseous compartments of the hand [5,6]. Gong and Lu report that the incidence rate of ACS in multiple metacarpal fracture is estimated to be 1.8% [7]. Rubinstein et al. report that, regardless of the severity of the ACS, outcomes for patients can vary from minimal hand dysfunction to amputation or even loss of life, due to tissue necrosis and cellular death [1]. The aim of this study is bimodal: firstly, to present two cases admitted in the emergency department of our unit, with multiple metacarpal fractures and developed ACS of the hand; and secondly, to create high clinical suspicion of this kind of lesions in physicians in the emergency department with scope to the early diagnosis and treatment.

## Case presentation

Case 1

**Figure 1:**
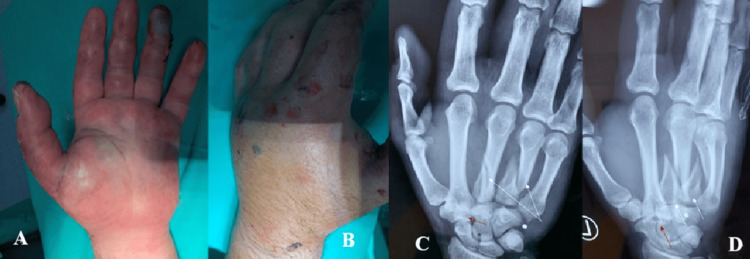
(A) Palmar and (B) dorsal surface depict the great swelling of the hand. Preoperative (C) anteroposterior and (D) oblique X-ray views show fractures of the third and fourth metacarpals (white arrows) and a fracture-dislocation of the base of the second metacarpal (red arrow).

A 28-year-old man was admitted to our department following a traffic accident (high-speed impact of a motorcycle and crush injury), and clinical examination revealed significant edema, particularly on the volar side of his left hand (Figures 1A, 1B). The patient reported severe pain during the active motion of the digits, although the capillary refill was normal. Radiographic examination revealed fractures of the third and fourth metacarpals, as well as a fracture-dislocation at the base of the second metacarpal (Figures 1C, 1D). Initially, the fractures were stabilized with a volar cast, and the patient was admitted to our department for surgical management of the injuries.

**Figure 2:**
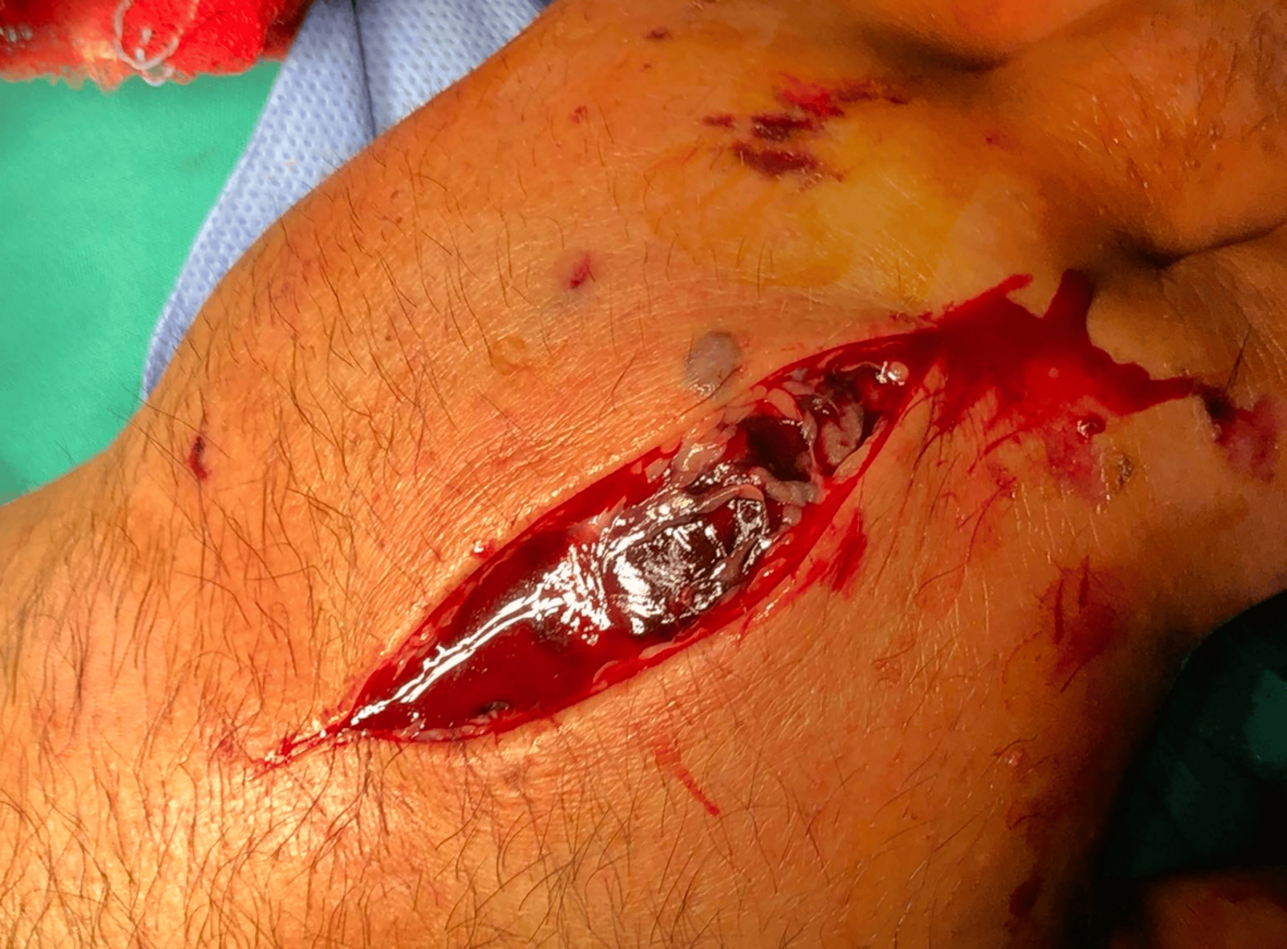
Hematoma on the dorsal surface of the hand (case 1).

After two hours, the patient reported severe pain during passive and active motions of the digits, paresthesia in the distribution of the median nerve, and impaired capillary refill through clinical examination. He was immediately taken to the theater, where, under general anesthesia, hand fasciotomies were performed (large hematoma with fluid was drained especially from the incision over second metacarpal which originated from interosseous muscles), while fractures were reduced and temporarily stabilized using Kirschner wires (Figure 2). The patient was immediately relieved of pain postoperatively and did not report any numbness in the hand. Within the next 10 days, gradual closure of the wounds was achieved, followed by definitive fixation of the fractures.

Case 2

**Figure 3:**
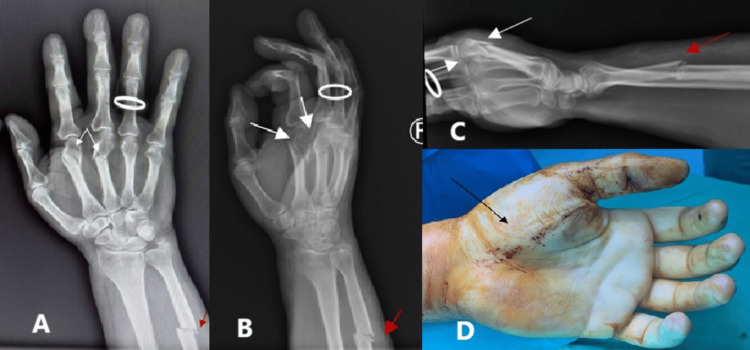
Preoperative (A) anteroposterior, (B) oblique, and (C) lateral X-ray views show fractures of the second and third metacarpals (white arrows) and a fracture of the distal ulna (red arrow). (D) The palmar surface shows significant swelling in the thenar region of the right hand (black arrow).

A 54-year-old man was transferred to our department from another unit three hours after falling from a height, caused fractures of the second and third metacarpals and the distal ulna of his right hand, as confirmed by radiographic examination (Figures 3A-3C). Clinical examination confirmed progressive swelling, particularly in the thenar region of the right hand (Figure 3D). The patient reported severe pain during passive motion of the digits, paresthesia in the distribution of the median nerve, and impaired capillary refill.

**Figure 4:**
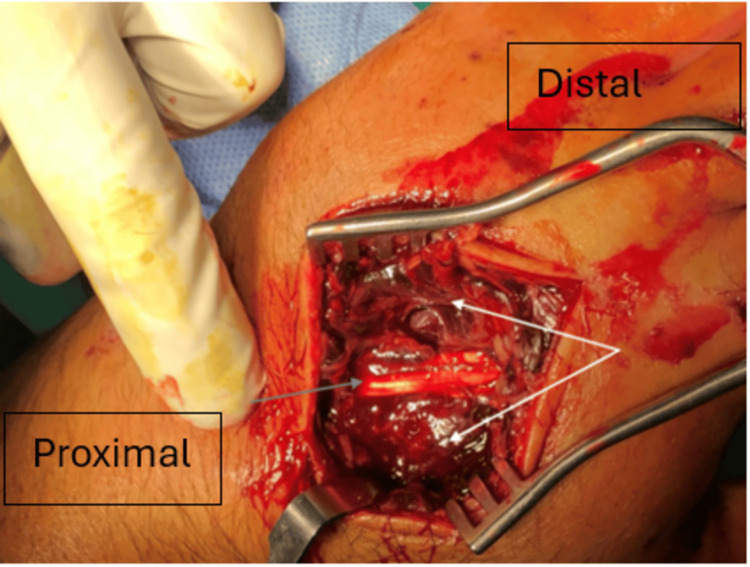
Intraoperative view from fasciotomy over the second metacarpal shows a large hematoma between the interosseous muscles (white arrows) and the extensor tendon of the index finger (blue arrow).

The patient was urgently admitted to the theater, where, under general anesthesia, hand fasciotomies (large hematoma was drained over hypothenar region) were performed (Figure 4). Postoperatively, the pain and paresthesia gradually subsided. Secondary closure of the surgical wounds was performed within the next 10 days, followed by definitive fixation (open reduction and internal fixation) of the fractures.

## Discussion

ACS of the hand is caused by progressively high pressure within the tightly confined myofascial compartments. Pathophysiologically, as interstitial pressure increases, capillary perfusion pressure is compromised, leading to a narrowed arteriovenous perfusion gradient that reduces muscle perfusion to the level necessary for cellular viability [2]. This ultimately results in hypoxia, nerve dysfunction, and muscle necrosis [1].

ACS has been reported in case reports or small case series, and a wide range of potential causes has been proposed. Alsaedi et al. conducted a systematic review and classified the causes of HCS into three categories: soft tissue injury (86.8%), including burns, animal bites, narcotic overdose, and crush injuries; fractures (5.4%), including multiple metacarpal fractures, with or without carpal bone fractures, and with or without fractures of the distal radius or ulna; and vascular injuries (7.8%), such as those involving the brachial or ulnar artery or potentially venous occlusion impairing outflow [5]. Dolan et al. presented a case of ACS that developed three hours after a traffic accident, which involved multiple fractures of the second, third, and fourth metacarpals [3]. Al-Qattan reported that three out of five cases of ACS in their study were associated with multiple fractures [6]. The authors mentioned that during dorsal fasciotomy, a significant hematoma was identified in the interosseous compartments [3,6,8]. In our cases, large hematomas were found in the first interosseous compartment due to the fractures, leading us to postulate that this was the primary cause of the progressive swelling that resulted in HCS.

Diagnosis is primarily based on clinical signs, while direct measurement of intercompartmental pressure provides objective support for diagnosing clinically suspected HCS [2]. Reichman established the "5 Ps" to describe the clinical symptoms of CS: pain, paresthesia, pallor, paralysis, and pulselessness [2]. However, the primary goal is early diagnosis, as waiting for the development of all clinical signs and symptoms could lead to permanent and severe sequelae [1,2]. Codding et al. suggest that the initial diagnosis should be based on the physician's suspicion (mechanism of injury and clinical presentation), while Rubinstein et al. noted that the most common clinical finding in ACS is a strained, swollen hand with intrinsic minus posturing [1,9]. The most common symptom of HCS is severe pain, regardless of the cause of injury. This pain is aggravated when the involved muscles are passively stretched and often requires increased analgesic administration [1,6]. In our cases, the primary symptoms included passive pain and extreme swelling of the hand, along with paresthesia in the distribution of the median nerve (particularly in the second case). Based on these clinical symptoms, we proceeded with hand fasciotomies.

Nevertheless, in some cases where clinical symptoms cannot be sufficiently evaluated to confirm a diagnosis of ACS (such as in intubated patients), measuring intracompartmental pressure becomes necessary [5,10]. In general, there is no consensus regarding the pressure threshold that indicates the need for surgical treatment: an absolute pressure of 15-25 mmHg, accompanied by clinical symptoms, or 25 mmHg without symptoms, is indicative of HCS [1,9,11]. Hand fasciotomy involves multiple incisions to facilitate access to all hand compartments and release of the carpal tunnel [1,2,9].

## Conclusions

ACS of the hand is a rare, multifactorial pathological condition, and its clinical presentation requires special attention. Our cases illustrate the necessity of maintaining high clinical suspicion for ACS in patients with multiple fractures of the hand in the emergency department. Physical examination and symptoms are the primary clinical characteristics of the diagnosis of the syndrome, and for this reason, we strongly recommend the hospitalization of patients, particularly if they lack the ability to obtain prompt follow-up or return to the emergency department, in order to closely monitor the hand for the potential development of ACS.
